# *Treponema pallidum* PCR screening at mucosal sites of asymptomatic men who have sex with men taking HIV pre-exposure prophylaxis

**DOI:** 10.1128/spectrum.00794-23

**Published:** 2023-09-06

**Authors:** Ei T. Aung, Christopher K. Fairley, Deborah A. Williamson, Francesca Azzato, Rebecca Wigan, Julien Tran, Andrew Buchanan, Tina Schmidt, Eric P. F. Chow, Marcus Y. Chen

**Affiliations:** 1 Melbourne Sexual Health Centre, Alfred Health, Melbourne, Victoria, Australia; 2 Faculty of Medicine, Nursing and Health Sciences, Central Clinical School, Monash University, Melbourne, Victoria, Australia; 3 Department of Infectious Diseases, The University of Melbourne, The Peter Doherty Institute for Infection and Immunity, Melbourne, Victoria, Australia; 4 Victorian Infectious Diseases Reference Laboratory, Melbourne, Victoria, Australia; 5 Centre for Epidemiology and Biostatistics, Melbourne School of Population and Global Health, The University of Melbourne, Melbourne, Victoria, Australia; Quest Diagnostics, San Juan Capistrano, California, USA

**Keywords:** MSM, syphilis, *Treponema pallidum*, PCR, polymerase chain reaction tests, syphilis screening, nucleic acid amplification tests, NAAT, PrEP, pre-exposure prophylaxis

## Abstract

Early detection and treatment of syphilis will reduce the infectious period and transmission. We aimed to determine whether screening men who have sex with men (MSM) taking HIV pre-exposure prophylaxis (PrEP) for syphilis using *Treponema pallidum* polymerase chain reaction (PCR) could detect syphilis before the appearance of syphilis antibodies in serology. MSM attending 3-monthly PrEP clinic visits in Melbourne, Australia, were screened with a PCR assay targeting the *pol*A gene of *T. pallidum* from an anal swab and an oral rinse between November 2019 and March 2020. Participants were serologically screened for syphilis using chemiluminescence immunoassay. A total of 309 asymptomatic participants provided an anal swab and oral rinse sample for *T. pallidum* PCR screening. Two syphilis cases (0.6%) were detected: one man had a positive serology only; another man had *T. pallidum* detected by PCR from an anal swab and a positive serology. PCR positivity was 0.3% (*n* = 1) for anal swabs and 0% (*n* = 0) for oral rinse. In this study, *T. pallidum* PCR screening at routine PrEP clinic visits did not identify additional cases of early syphilis over serological screening performed at these visits.

**IMPORTANCE:**

With the ongoing syphilis epidemic in men who have sex with men (MSM), we investigated the role of using *Treponema pallidum* polymerase chain reaction (PCR) testing at the oral cavity and anus in MSM taking pre-exposure prophylaxis for the early detection of syphilis. We evaluated whether the PCR tests from these mucosal sites can detect syphilis infection early, before the development of syphilis antibodies in serology. Our study found two syphilis cases among 309 MSM, and only one syphilis case had a positive anal PCR swab, although serology was positive. We conclude that additional PCR testing is likely to be expensive and would not be cost effective for individuals who regularly screen for syphilis. However, future studies with a larger sample size are required.

## INTRODUCTION

Gay, bisexual, and other men who have sex with men (MSM) have a high prevalence of syphilis, with an estimated global prevalence of about 8% (1), higher than the prevalence in the general population. In Australia, MSM are disproportionately affected by syphilis (2, 3). Longstanding public health interventions aimed at controlling syphilis, such as serological screening and contact tracing and treatment, have not curtailed growing syphilis epidemics among MSM internationally, warranting novel interventions (4 – 7).

In many high-income countries, 3-monthly screening for sexually transmitted infections (STIs) has been recommended for MSM taking HIV pre-exposure prophylaxis (PrEP) (8). Despite an increase in coverage and frequency of serological screening in Australia, syphilis incidence remains high among MSM (9) with the highest rates occurring in MSM taking HIV PrEP (10). The incidence of syphilis among MSM taking HIV PrEP was reported to be between 8 and 13 per 100 person-years (i.e., incidence rate between 8 and 13 syphilis diagnoses in 100 person per year). (10 – 12). A previous study on MSM taking HIV PrEP in Melbourne showed that a significant proportion of primary and secondary syphilis was detected between the routine 3-monthly PrEP monitoring visits, suggesting that 3-monthly serological screening for syphilis alone is unlikely to be sufficient to reduce syphilis transmission in this high incidence population (13). Moreover, the use of serology for screening has the potential to miss a proportion of primary syphilis infections. In a study analyzing 814 primary syphilis cases, 5% (*n* = 38) of cases detected by *Treponema pallidum* polymerase chain reaction (PCR) had negative syphilis serology (14). The finding implies that *T. pallidum* PCR might be able to detect syphilis infection early before a positive syphilis serology. Early detection of syphilis infection is required to reduce the duration of infectious period and reproductive rate of syphilis.

Nucleic acid amplification tests (NAAT) such as PCR for *T. pallidum* are highly sensitive and specific for the diagnosis of primary syphilis (15 – 18). To date, most studies have examined the use of *T. pallidum* PCR for diagnosing syphilis from suspected early lesions rather than for screening (17 – 21). In these studies, the detection of syphilis depends on the clinically evident syphilis lesions. However, primary syphilis lesions can occur in the oral cavity and anal canal where they might be hidden and not clinically evident (22). Furthermore, unrecognized oral and anal shedding with or without lesions in the oral cavity and anus have been shown to occur in early syphilis (23).

Studies have shown that *T. pallidum* can be detected by PCR from oral and anal samples of MSM with early syphilis with or without symptoms (23 – 25). Oral detection was most frequent in those with secondary syphilis (23), although syphilis detection by PCR occurred in various sample types in all early stages of syphilis in the absence of lesions (25). Based on these findings, we hypothesized that PCR screening from the oral cavity and anus might detect syphilis before the appearance of syphilis antibodies in serology in the absence of syphilis lesions.

In this study, we aimed to determine whether screening MSM taking HIV PrEP using *T. pallidum* PCR from oral and anal samples could detect syphilis before they were serologically positive. We chose MSM taking HIV PrEP as the study population due to the high incidence of syphilis in this group.

## MATERIALS AND METHODS

This was a cross-sectional study of MSM attending the HIV PrEP clinic at the Melbourne Sexual Health Centre, Australia, between November 2019 and March 2020. Men attending the clinic were reviewed every 3 months to screen for HIV and STIs and to obtain their PrEP medication. The clinic has an electronic medical record system that collects demographic and epidemiological data. In this study, we defined MSM as gay, bisexual, and other men who had sex with men in the last 12 months. We included transgender women taking PrEP who were having sex with men.

Individuals who attended to the HIV PrEP clinic for their 3-monthly review were asked to self-collect an anal swab and an oral rinse specimen for *T. pallidum* PCR testing. Self-collection was chosen as the preferred method, as the majority of clients attending the HIV PrEP clinic were regular attendees and were already familiar with self-collecting swabs for chlamydia and gonorrhea. Moreover, past studies have demonstrated comparable sensitivity between self-collected and clinician-collected swabs (26, 27). Eligible individuals were given a participant information sheet explaining the study, and verbal consent was obtained from the participants. The anal swab was collected using ESwab (Copan Diagnostics Inc., Murrieta, CA, USA). An oral rinse was collected by gargling 10 mL of sterile water for injection (Pfizer, Bentley, WA, Australia) and swilled for 15 s.

### DNA extraction

Total DNA was extracted from fresh specimens on the Freedom EVO 100 (Tecan, Mannedorf, Switzerland) using the Quick DNA/RNA MagBead Extraction Kit (Zymo Research, Irvine, California, USA), as per the manufacturer’s instructions. DNA extracts were stored at −20°C, and the original lesion and non-lesion study samples were stored at −70°C.

### PCR testing

Specimens were tested using a TaqMan real-time PCR assay targeting the *polA* gene of *T. pallidum,* which was previously validated for clinical use with lesion samples (28). Testing was performed at the Victorian Infectious Diseases Reference Laboratory, Melbourne, Australia (28). Details of the *T. pallidum* PCR testing are described elsewhere (28). For a positive *T. pallidum* PCR sample, a cycle threshold (Ct) of 38 was determined as a cut-off value. If the Ct value was greater than or equal to 38, a repeat testing was performed, and the result was reported as positive if the serology was also positive. The *T. pallidum* PCR assay in our study has a sensitivity of 80% and specificity of 98% for detecting early syphilis infection from lesion samples (28). The limit of detection was determined to be one DNA copy per microliter for this PCR assay. The limit of detection was determined through a process of a serial dilution using a standard control, known as Amplirun Treponema Control (Vircell Microbiologists, Madrid, Spain).

Serology for syphilis was obtained at each 3-monthly clinic visit with testing using chemiluminescence immunoassay (CLIA) (Diasorin, Saluggia, Italy), with confirmation by *T. pallidum* particle agglutination assay (TPPA) (Fujirebio, Tokyo, Japan) and rapid plasma reagin (RPR) (Becton Dickinson, New Jersey, USA). Routine screening of chlamydia and gonorrhea with NAAT was performed using Aptima Combo 2 assay (Hologic Panther system; Hologic Inc., San Diego, CA, USA) on urine specimens, pharyngeal swabs, and anal swabs, if collected. HIV screening was performed using the DiaSorin Liaison XL Murex HIV Ab/Ag CLIA (fourth generation) assay. Separate anal swabs were obtained for *T. pallidum* PCR and chlamydia/gonorrhea NAAT.

A syphilis diagnosis was defined as testing positive by PCR or serology or both (29). The Australian Department of Health Public Health Laboratory Network defined an active syphilis infection as having reactive non-treponemal tests or demonstrated seroconversion of treponemal test within 12 months of a negative test or detection of *T. pallidum* by NAAT (i.e., positive PCR) (29). For participants with a history of syphilis, an active infection was defined as having more than a fourfold rise in RPR titer and/or a positive *T. pallidum* PCR. In cases where only treponemal-specific serology was positive (i.e., positive CLIA and TPPA) without a reactive RPR, positive *T. pallidum* PCR, or any signs and symptoms of syphilis, the clinical team would confirm the participants’ past infection with syphilis and treatment history with the participants in order to determine the presence of an active syphilis infection and to stage the infection correctly.

Categorical variables were reported as frequencies and percentages, and continuous variables as median and interquartile range (IQR). The number needed to screen to obtain a positive *T. pallidum* PCR test (i.e., indicating a new syphilis infection) was calculated by dividing the number of participants tested for syphilis by the number of participants tested positive for syphilis (30). This calculation was performed separately for anal swab and oral rinse samples. We also calculated the 95% confidence intervals (CIs) for syphilis diagnoses using binomial proportion confidence interval. We anticipated to recruit 1,200 participants over the 36-month period of evaluation. The study was stopped prematurely due to the COVID-19 pandemic in March 2020. During the analysis, we performed a chart review to confirm that the participants included in the study were unique patients. This step was taken to align with the cross-sectional design of the study. All analyses were performed using Stata (Version 16, Texas, USA).

## RESULTS

Between November 2019 and March 2020, 309 individuals taking HIV PrEP attended the PrEP clinic for their regular 3-monthly PrEP review appointments and consented to provide both anal swab and oral rinse specimens for *T. pallidum* screening and were included in the analysis.

The median age was 34 years (IQR: 29–42), and the median number of male sexual partners in the last 3 months was 15 (IQR: 7–23). Most participants (92%, *n* = 285) had condomless anal sex in the last 3 months. Thirty-two percent (*n* = 92) had a past history of syphilis infection. Most participants (98%, *n* = 303) were taking daily PrEP, with 4% (*n* = 12) reported taking doxycycline as prophylaxis for STIs in the last 1 month. None presented with signs and symptoms of syphilis at presentation or as a sexual contact with an individual with syphilis infection. Table 1 shows the characteristics of the 309 participants.

**TABLE 1:** Demographics, sexual practices, and medical history of 309 participants taking HIV PrEP

	Participants (*n*)	Percentage
Age [median (IQR)]	34 (29–42)	
Gender		
Male	306	99
Transgender women	2	0.7
Other	1	0.3
Sexual practice in the last 3 mo		
Sex with men only	298	96
Sex with men and women	11	4
Male partners in the last 3 mo [median (IQR)]	15 (7–23)	
Intravenous drug use		
Never	289	94
≤12 mo	14	4.5
>12 mo	2	0.7
Unknown	4	1.3
Condomless anal sex with male partners in the last 3 mo		
No	23	7
Yes	285	92
Unknown	1	0.3
Past history of syphilis		
No	210	68
Yes	99	32
HIV PrEP use		
Daily	303	98
Event driven	6	2
Doxycycline prophylaxis in the last 1 mo		
No	297	96
Yes	12	4

### Syphilis diagnosis

Two men (0.6%, 95% CI: 0.1%–2.3%, 2/309) were diagnosed with infectious syphilis. One man had positive syphilis serology and negative *T. pallidum* PCR results. The other man had positive syphilis serology and *T. pallidum* detected by PCR from the anal swab. The positivity of *T. pallidum* PCR was 0.3% (95% CI: 0.01%–1.79%, 1/309) for anal swabs and 0% (95% CI: 0.0%–1.2%, 0/309) for oral rinse.

The man with positive syphilis serology and anal *T. pallidum* PCR had 40 male sexual partners in the last 3 months and did not report symptoms or signs of syphilis, including at the anus. His RPR titer was 1:8, which increased from 1:1 when the serology was performed 3 months earlier. Six months prior to the current consultation, he had received treatment for early latent syphilis infection with an RPR titer of 1:16 at that time. *T. pallidum* PCR from the anal swab had a Ct value of 37. He was also diagnosed with anal and pharyngeal gonorrhea. The other man with positive syphilis serology alone had nine male sexual partners in the last 3 months, did not have symptoms or signs of syphilis, and had no past history of syphilis. The RPR titer was 1:8 and *T. pallidum* PCR was negative from both the anal swab and the oral rinse.

### Number needed to screen for syphilis detection using *T. pallidum* PCR

We estimated that the number needed to screen to detect a syphilis infection using *T. pallidum* PCR testing on an anal swab was 309. However, we were unable to calculate the number needed to screen for syphilis using an oral rinse, as we did not obtain any positive results from the oral rinse samples.

### Estimated cost of using *T. pallidum* PCR for syphilis detection

The estimated cost of one *T. pallidum* PCR test for syphilis detection is AUD $35, excluding transport and processing costs (31). In our study, we utilized two *T. pallidum* PCR tests—anal swab and oral rinse—resulting in an additional cost of AUD $70 per person per visit, in addition to the syphilis serology test. The cost per additional syphilis case detected using additional *T. pallidum* PCR tests is estimated to be $21,600, calculated based on the need to test at least 309 individuals to detect a single case of syphilis, multiplied by the cost of $70 per person.

### STI diagnoses

Bacterial STIs were diagnosed in 16% (48/309) of the participants. The positivity of chlamydia was 8% (24/309), with the most common infection in the anus (6%, 20/309), followed by the urethra (2%, 6/309), and the pharynx (0.3%, 1/309). The positivity of gonorrhea was 8% (24/309), with the most common infection in the pharynx (5%, 16/309), followed by the anus (4%, 12/309) and the urethra (0.6%, 2/309).

## DISCUSSION

We conducted this study to examine the role of *T. pallidum* PCR screening of the oral cavity and anus among individuals at high risk of syphilis infection. We recruited 309 asymptomatic individuals taking HIV PrEP who had previously been reported to have a relatively high incidence of syphilis. Only one case of asymptomatic rectal *T. pallidum* was detected, and this case had a positive syphilis serology. No participant tested positive by PCR from the oral cavity. Our data suggest that using our method for *T. pallidum* PCR screening of the oral cavity and anus among MSM taking PrEP may not be cost effective. Our data are consistent with a previous study showing that PCR testing is unlikely to identify a significant number of cases earlier than serology (32).

To our knowledge, our study is the second study to examine the role of *T. pallidum* PCR screening of asymptomatic MSM at the mucosal sites. A previous study conducted in the USA found that 0.4% (2/441) of MSM tested positive on anal swab by both transcription-mediated amplification (TMA) testing and PCR, targeting the *tpp47* gene before seroconversion of syphilis antibodies (32). In contrast, in our study, the individual with a positive anal PCR swab also had a concurrent positive syphilis serology. Furthermore, in the US study, one individual with a positive anal PCR in the absence of a positive serological test was a syphilis contact with a rectal ulcer, and therefore, the risk of syphilis infection would have been much higher. In our study, none of the syphilis cases was a syphilis contact. It is uncertain whether *T. pallidum* PCR screening of MSM who are syphilis contacts might detect syphilis by PCR before serology.

Whether or not the detection of *T. pallidum* by PCR from oral or anal sites precedes the appearance of syphilis antibodies will depend on several factors. This includes the sensitivity of the PCR and serological assays used in the detection of very early asymptomatic infection. The in-house real-time *T. pallidum* PCR assay in our study has a sensitivity of 80% and specificity of 98% for detecting early syphilis infection from lesion samples (28). The assay has not been validated for non-lesion samples, and therefore, the sensitivity might be lower than the lesion samples. Moreover, when comparing *T. pallidum* PCR to TMA, the sensitivity of *T. pallidum* PCR was slightly lower with PCR positive in eight of nine (89%) TMA-positive rectal specimens (32). It is possible that the number of syphilis cases detected by PCR was lower in our study because of the less sensitive *T. pallidum* molecular assay. Additionally, the two molecular assays target different regions of *T. pallidum,* with TMA targeting 23S rRNA and PCR in our study targeting *polA* gene of *T. pallidum,* respectively. Another factor to consider is the use of CLIA as a serological screening test, which has been shown to have excellent sensitivity and specificity as a confirmatory and a screening test for syphilis (33). In a comparative study where CLIA and enzyme immunoassay (EIA) were evaluated, using TPPA as a confirmatory test, CLIA demonstrated higher sensitivity than EIA (94% vs 83%) (34). However, it remains uncertain whether CLIA assays are more sensitive than other immunoassays. If CLIA assays were to exhibit earlier reactivity than other serological assays, this could potentially diminish any additional advantages from *T. pallidum* PCR screening.

It is unlikely that adding *T. pallidum* PCR as a screening test would be cost-effective. An Australian study estimated AUD $35 per *T. pallidum* PCR test, not including transport and processing costs (31). That means an estimate of an extra AUD $70 per person per visit with two *T. pallidum* PCR tests added to the existing syphilis serology tests, which cost around AUD $15 (35). Our data suggest that the cost per extra case detected would exceed $20,000, and given that these individuals have serology every 3 months, it means infections are missed for a relatively short period of time. This calculation is dependent on the incidence of syphilis and the proportion of early cases that are PCR positive and serology negative. With a recommendation of 3-monthly STI testing for men taking HIV-PrEP, the costs for syphilis screening would rise if *T. pallidum* PCR tests were integrated into routine STI screening.

There are several other limitations that should be noted. First, we only recruited 309 participants in this cross-sectional study with very few men who tested positive for syphilis. Our initial protocol was to recruit 1,200 men for over 36 months; however, the study was stopped after 5 months due to the COVID-19 pandemic and lockdown in Victoria, Australia. A small sample size could affect the accuracy of the results. Second, the study period was short, and this could underestimate the incidence of syphilis. Third, none of the participants presented as a syphilis contact, who are at higher risk for syphilis infection. Fourth, it is possible that some participants may have had syphilis diagnosed elsewhere, such as general practice, between the regular review appointments for HIV PrEP. Fifth, it is possible that not all clinic attendees participated to perform *T. pallidum* screening tests, which could have underestimated the number of syphilis infections. We did not have the data on those who declined the additional screening tests. Last, we included only MSM and transwomen taking PrEP, whose risk of syphilis infection and STI screening practice might differ from other MSM or transwomen who were not taking PrEP or not regularly screened. Moreover, the findings might not be generalizable to heterosexual men and women who might not have STI screening as frequently as MSM. The value of PCR screening in heterosexual populations, particularly in high-risk women, should be evaluated in light of re-emergence of congenital syphilis and an increase in syphilis cases in heterosexual men and women (3, 36).

Because of the limitations of our study, larger studies of high-risk populations, not limited to MSM on HIV PrEP, using more sensitive *T. pallidum* assays for screening are warranted. These studies should be accompanied by cost-effectiveness analyses to determine if the cost of NAAT screening is offset by earlier diagnosis and reductions in syphilis transmission and sequelae.

## Data Availability

The data are not publicly available due to privacy and ethical restrictions, however, will be made available on reasonable request from the corresponding author, with the permission of the Alfred Hospital Ethics Committee. Restrictions apply to the availability of these data, which were used under license for this study.
